# Extracellular detection of neuronal coupling

**DOI:** 10.1038/s41598-021-94282-6

**Published:** 2021-07-19

**Authors:** Elmer Guzman, Zhuowei Cheng, Paul K. Hansma, Kenneth R. Tovar, Linda R. Petzold, Kenneth S. Kosik

**Affiliations:** 1 Department of Molecular, Cellular and Developmental Biology, University of California, Santa Barbara, Santa Barbara, CA USA; 2 Neuroscience Research Institute, University of California, Santa Barbara, Santa Barbara, CA USA; 3 Department of Computer Science, University of California, Santa Barbara, Santa Barbara, CA USA; 4 Department of Physics, University of California, Santa Barbara, Santa Barbara, CA USA

**Keywords:** Neurophysiology, Data processing

## Abstract

We developed a method to non-invasively detect synaptic relationships among neurons from in vitro networks. Our method uses microelectrode arrays on which neurons are cultured and from which propagation of extracellular action potentials (eAPs) in single axons are recorded at multiple electrodes. Detecting eAP propagation bypasses ambiguity introduced by spike sorting. Our methods identify short latency spiking relationships between neurons with properties expected of synaptically coupled neurons, namely they were recapitulated by direct stimulation and were sensitive to changing the number of active synaptic sites. Our methods enabled us to assemble a functional subset of neuronal connectivity in our cultures.

## Introduction

MEAs can monitor and record extracellular action potentials (eAPs, also known as 'spikes') from hundreds of neurons simultaneously. However, because MEA electrodes measure voltage changes from the extracellular electric field, eAPs from multiple neurons can be detected by single electrodes. This limitation means that reliably assigning a specific neuron as the source of any action potential is typically not feasible. Analyzing the relationships between eAPs at different MEA electrodes requires assigning spikes to different source 'units' through the process of spike sorting^1^. Many spike sorting algorithms exist but many of these methods do not incorporate ground truth validation of experimental data^2,3^ and therefore it is not possible to routinely assess the validity of spike sorting outputs.

We recently developed an empirical approach to unambiguously identify eAPs from single neurons, based on detection of axonal action potential propagation by multiple extracellular electrodes^4^. When propagating eAPs are detected by multiple electrodes along the propagation path, those spikes are unambiguously self-classified as originating from single neurons. The electrodes in each propagation cohort, the order of eAP occurrence and the eAP latency between each electrode are features that identify each signal. We refer to the action potentials detected by these electrode cohorts as propagation signals. Multiple unique propagation signals are present on each array in our experiments. Here we examined the intercellular relationships between single cultured mouse hippocampal neurons isolated by their distinctive propagation signals and eAPs at all other array electrodes.

Superimposing the eAPs from constituent propagation signal electrodes corresponding to each unique neuron effectively recalibrates the timing of the voltage record from all other array electrodes to the propagation signal spike times. Isolating spiking from single identified neurons in this way revealed clusters of spikes at other electrodes occurring within milliseconds of the preceding propagation signal spike. We studied the underlying nature of these associations in spike timing by using stimulation, by decreasing the probability of neurotransmitter release and by changing the recording temperature. Our results are consistent with direct synaptic coupling underlying many of the short latency relationships between propagation signal spikes and the spike clusters at other electrodes. The amplitude distributions of the coupled eAP clusters are often statistically discrete subsets of the full eAP amplitude distribution at those electrodes, consistent with coupled spikes representing eAPs from single postsynaptic neurons. Our results suggest that in our in vitro system, axons can make a sufficient number of synapses on postsynaptic neurons to result in firing of those neurons. Our methods can be generalized to reveal in vitro network connectivity phenotypes from targeted mutant or iPS-derived neurons that might be overlooked by more traditional experimental approaches.

## Results

### Propagating eAPs are a timing device

**Figure 1:**
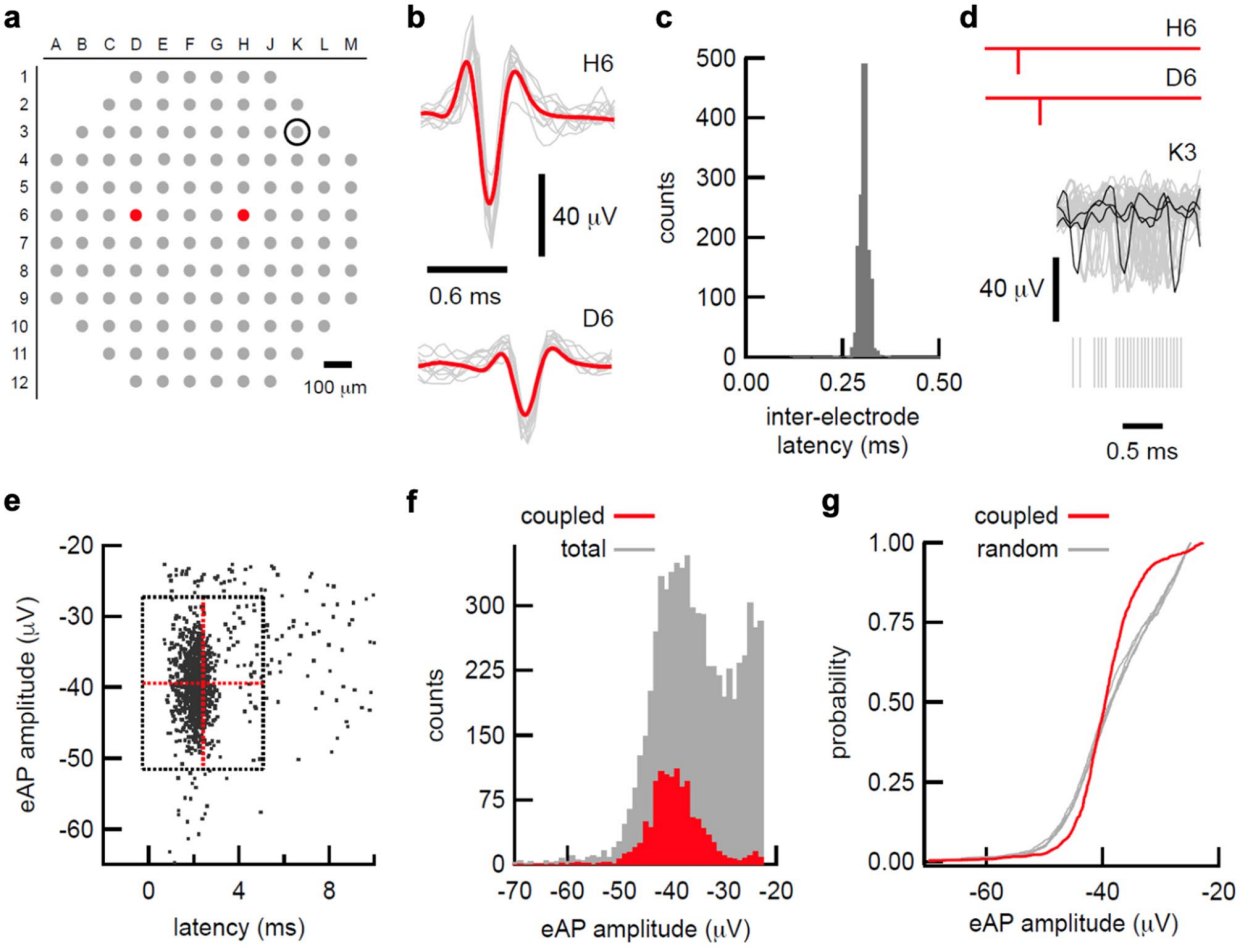
Propagation signals are a clock used to identify coupled eAP’s. (**a**) Map of electrode array used in these experiments. Two electrodes that detect single axon eAP propagation are shown in red. Electrode K3 is the location of the spike cloud that follows the propagation signal. (**b**) The eAP waveforms at H6 and D6 are shown. Individual eAPs are in grey; the average of 100 eAP waveforms is superimposed in red. (**c**) The distribution of inter-electrode eAP latency between H6 and D6. The high number of co-occurrences (n = 1360) and low coefficient of variation (0.078) are consistent with action potential propagation. (**d**) aligning the eAP co-occurrences at H6 and D6 (schematized in red) reveals a cloud of eAPs at K3. Twenty eAP waveforms from K3 are superimposed in grey, with 3 waveforms highlighted in black. The timing of 50 eAPs from K3 following the H6/D6 co-occurrence are shown as grey hash marks beneath eAP the waveforms. (**e**) the plot of eAP amplitude at versus time after H6/D6 co-occurrence shows a large cluster. All eAPs that occurred between 0.5 and 10 ms after the H6/D6 co-occurrence are displayed. Red lines indicate the mean latency and eAP amplitude, respectively. Horizontal and vertical grey lines indicate two standard deviations of the mean for each dimension. (**f**) the amplitude distribution of the spikes in K3 that occurred within 0.5 to 10 ms following the H6/D6 co-occurrence (red bars) are superimposed on the all-points spike amplitude distribution histograms from electrode K3. (**g**) cumulative distribution of the coupled spikes in K3 (red line) superimposed on the cumulative distributions from 5 randomly selected groups of amplitudes from the all-points distribution from K3, showing that the amplitudes of the coupled eAPs were not randomly selected from the full amplitude distribution.

We isolated eAPs from single identified neurons by extracting action potential propagation signals from single axons across multiple electrodes^4,5^. We previously characterized axonal action potential propagation by isolating cohorts of electrodes with eAPs that occur in fixed spatial and temporal relationships^4^ as a means to identify and label each individual isolated neuron. For example, spikes between electrodes H6 and D6 (Fig. 1a,b) co-occurred 1360 times in this recording, with an inter-electrode latency of 0.305 ± 0.024 ms and a coefficient of variation (CV) of 0.078, consistent with a high-fidelity process like axonal action potential propagation. Isolation of eAP propagation signals within the multi-electrode voltage record in this way empirically reveals spiking from single neurons.

The co-detection of action potentials by multiple electrodes was the time stamp we used to mark events at other array electrodes. Because co-occurrences represent spiking from a single neuron, we can recalibrate the timing of all other electrodes by indexing the voltage record to the co-occurring eAPs. Superimposing the co-occurring spikes from each unique propagation signal recalibrates the timing of other eAPs with relation to the timing of the propagating events. For example, superimposing co-occurring spikes from H6 and D6 (Fig. 1c) revealed a cluster of spikes at another electrode (K3) that occur with short, variable latency after the propagating spikes (Fig. 1d). In this example, spikes in K3 occurred with a probability of 0.85 between 0.5 to 10 ms after the propagating eAPs, with a latency of 2.40 ± 1.33 ms (n = 1205; Fig. 1e). The latency CV of these coupled spikes (0.55) is inconsistent with action potential propagation while the high probability and short latencies of eAPs in K3 suggested direct coupling of spiking between the propagation signal neuron and a neuron detected at electrode K3.

Extracellular electrodes can detect spikes from any sufficiently proximal neuron. Consequently, the eAP amplitude distribution of single electrodes can reflect spikes from multiple source neurons^6^; spike amplitude can often be discriminating criteria in spike sorting routines^2,7^. The full eAP amplitude distribution from electrode K3 is multimodal, potentially representing spikes from at least two source neurons (Fig. 1f, grey bars). In contrast, the amplitude distribution of the eAPs at K3 that occurred between 0.5 to 10 ms after propagation signal H6/D6 was a discreet subset of the entire eAP amplitude distribution rather than a randomly sampled subset of the full amplitude distribution (Fig. 1f, red bars). To formally examine if the amplitude distribution of coupled spikes at K3 could be explained by random selection from the entire amplitude histogram, we compared the amplitude distribution of the eAPs coupled to the propagation H6/D6 to the amplitude distributions of multiple randomly sampled eAPs amplitudes from K3, using the Kolmogorov–Smirnov (KS) test, which tests the probability that groups of spikes were taken from the same distribution (Fig. 1g). The probability that the distribution of coupled eAPs and randomly sampled eAPs were from the same distribution was less than 1.0 × 10^–14^ (two-sample KS test, two-sided, n = 1205). In contrast, the probability that two randomly chosen groups of eAPs from electrode K3 were drawn from the same amplitude distribution was 0.64. These results suggest that eAPs from a single source neuron occur with short latency after the propagating spike H3/E6. Our results further suggest the short latency between eAPs from the neuronal source of the propagation signal eAPs and downstream clusters of spikes reflects direct synaptic coupling.

### Detection of neuronal coupling with an automated detection algorithm

**Figure 2:**
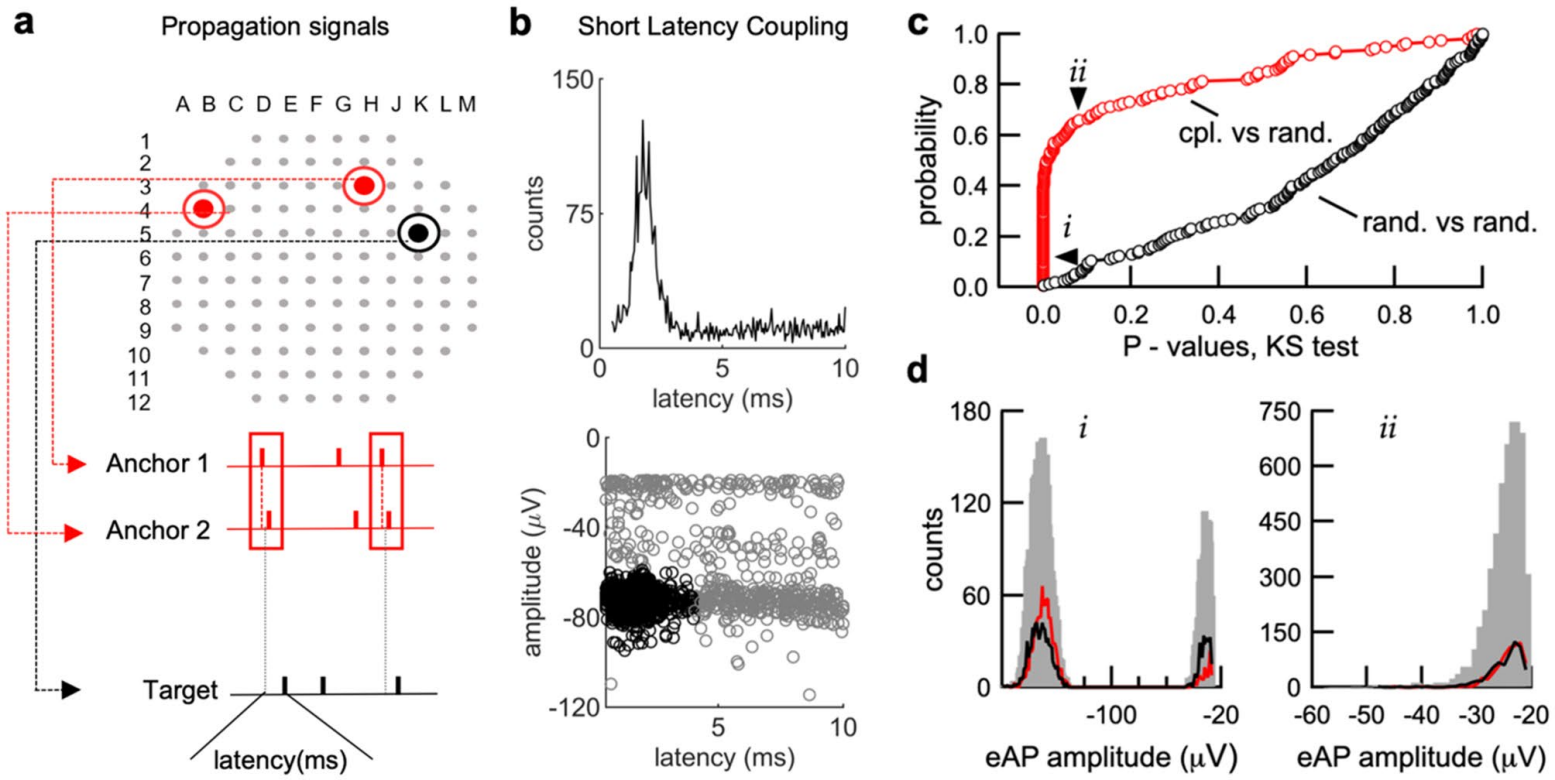
Algorithm for the routine detection of synaptic coupling. (**a**) following propagation signal detection, two electrodes with the most co-occurrences from each propagation signal were picked as anchor points to compute the spike times. (**b**) A CCG is generated and stored using propagation signal spike times as a reference and all other individual electrodes as targets. To inspect the specificity of the postsynaptic response, a scatter plot of spike amplitude versus spike latency in the target electrode is produced. A few criteria can be used for deciding the existence of a short latency connection including: 1) The position of the peak of the CCGs, 2) the standard deviation of the latency and amplitude, 3) the proportion of spikes that fall into the peak region, and 4) the ratio of the number of spikes in the peak region to the number of spikes in the reference signal. The specific values for the criteria can alternatively be user defined. (**c**,**d**) (right) The two-sample KS test (two-sided) *P* value distribution of coupled-versus-coupled comparisons (red circles) and random-versus-random comparisons (black circles). The amplitude distributions of some electrodes are expected to be unimodal if eAPs from only single neurons are detected by those electrodes. In these cases, coupled spikes would be expected to have the same amplitude distribution as randomly selected spikes, as is the case for comparisons that do not differ.

To apply the high throughput capabilities of MEAs efficiently, we developed algorithms for the automated detection of (1) propagation signals and (2) neurons with short latency couplings based on clusters of post-synaptic eAPs. We first isolated eAPs from single neurons, using all 120 electrodes to detect propagation signals. We then used the propagation signal spike times to create cross-correlograms (CCGs), with a 2 ms window before and after reference time-points for each pair of eAP spike time comparisons among all electrodes. The process was repeated for all array electrodes, generating a collection of all propagation signals with the delay time of the electrodes through which they pass (Fig. 2a).

The spike times of each propagation signal were then used as reference time-points for detection of short-latency connections between propagation signal spike times and eAPs at all other electrodes. A new CCG was performed for each pair of comparisons, using a window between 0.5 and 10 ms after each reference time-point (Fig. 2b) with filtering criteria described in the Methods section. The coupling probability is the ratio of spikes in the CCG peak over the total number of propagation signal spikes.

To test whether our algorithms were randomly selecting eAPs from each electrode, we used the two-tailed KS test to determine the probability that coupled spikes were selected from the same amplitude distribution as a randomly selected group of spikes from the entire distribution of the same electrode. We also compared two groups of randomly selected eAPs from the same electrode to test the probability that they were from the same distribution. As seen, most (94/156) of the coupled-versus-random comparisons (red circles) were unlikely to have been selected from the same distribution at a 5% significance threshold (Fig. 2c). In contrast, 152 of the random-versus-random comparisons (black circles) exceeded this threshold. These data indicate that the coupled spikes selected by our algorithm are a non-random selection from the full eAP amplitude histogram at each electrode.

The amplitude distribution of coupled spikes (red lines) selected by our algorithm in this example (Fig. 2d, left) are predominantly sampled from the higher amplitude mode of the full distribution (grey bars). In contrast, the randomly drawn sample of eAP amplitudes (black line) mirrors the full amplitude distribution. This difference led to a low probability (*P* < 6.7 × 10^−35^) that coupled spikes and randomly selected spikes were drawn from the same sample. However, in some cases, such as when only one neuron is proximal to an electrode, the amplitude distribution will approximate a normal distribution. Thus, when we apply our algorithm under these circumstances, the coupled spike amplitude distribution will be indistinguishable from a randomly selected group of spikes (Fig. 2d, right). In cases where electrodes sample from multiple neurons, coupled spikes extracted by our algorithm represent a statistically distinct subset of the full events, consistent with eAPs from single neurons and with statistical characteristics outlined previously (Fig. 1f,g). To examine whether our results arose because of random associations between pre- and postsynaptic neurons, we shuffled the timing of all spikes at postsynaptic electrodes, while retaining the distribution of inter-spike intervals (ISI) for each electrode and calculated the ratio of the number of spikes in the target electrode that fall within 0.5 to 10 ms of a spike in the reference electrode over the number of spikes in the reference electrode signal. We found that the ratio dropped from 0.35 ± 0.25 in unshuffled controls to 0.0035 + − 0.0038 in the shuffled data (n = 746 detected couplings), indicating that what our algorithm identified as couplings were not the result of the randomness of timing associations between spikes at different electrodes. Our results indicate that our algorithm detects spikes that are postsynaptic to the propagation signal eAP, likely representing examples of synaptically-coupled neurons from the extracellular voltage record.

### Stimulation recapitulates properties of spontaneously-coupled spikes

**Figure 3:**
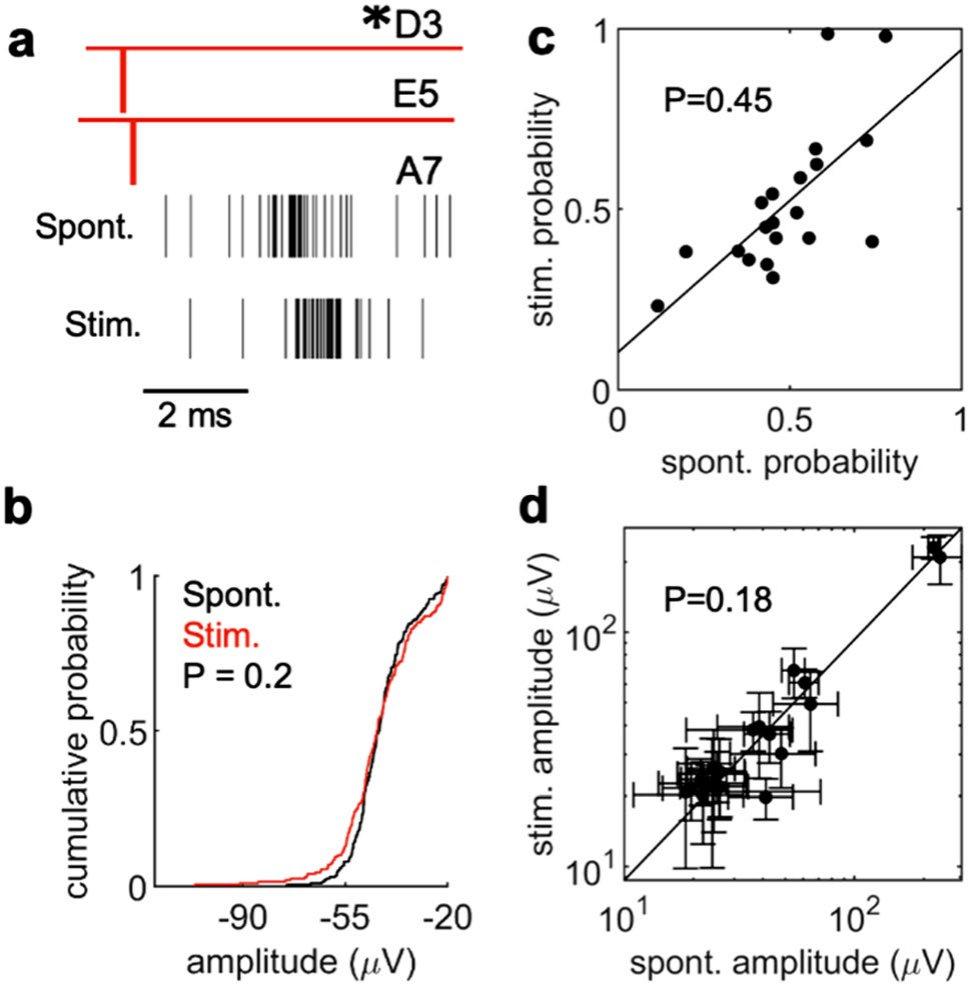
Stimulation of presynaptic neurons recapitulates spontaneous activity. (**a**) 50 spontaneous co-occurring spikes in D3 and E5 were used as reference points to identify coupled spikes in A7 (spikes depicted as raster’s below). Spiking activity in A7 after stimulation of electrode D3 using a 3µA biphasic current injection, 200 µsec total duration, 500 times. A blanking period (1.5 ms) during which no voltage data is collected is applied to all electrodes due to artifacts introduced by stimulation. For the stimulation experiments we therefore measured latency from the start of the stimulation period. (**b**) The spike amplitude distribution of spikes detected at A7 (n = 200 randomly sampled spikes) after spontaneous propagation signals at D3/E5 (n = 572) is not significantly different than the spike distribution of spikes detected at A7 (n = 200 randomly sampled spikes, *P* = 0.2, two-sample KS test, two-sided, 1 MEA with 2 recording sessions for (**a**,**b**)) after stimulation at electrode D3. (**c**) Correlation of coupling probabilities for spontaneous activity versus stimulated activity. Each data point in (**c**) and (**b**) represents coupled neurons. Couplings were identified by identifying spontaneous propagation signal activity as references in a CCG and a coupling probability was assigned to the postsynaptic unit. Following identification of coupling relationships, electrodes associated with presynaptic propagation signals were then stimulated in order to obtain coupling probabilities with the same postsynaptic unit (n = 20 couplings, *P* = 0.45 paired t-test). (**d**) For the same coupling events in (**c**), the correlation between spike amplitudes of the postsynaptic response in the spontaneous condition was compared to the spike amplitudes of the stimulated condition. Error bars represent the standard deviation of spike amplitude distributions (n = 20 couplings, *P* = 0.18 paired t-test, 3 MEAs and 6 total recording sessions were used for (**c**,**d**)).

The majority of eAPs in our cultures result, directly or indirectly, from excitatory synaptic transmission. Blocking excitatory neurotransmitter receptors in our cultures (2.5–5 µM NBQX and 10 µM R-CPP) reduced the number of spikes to 12.5 ± 15.2% of control (n = 7 arrays). The latency between propagation signal eAPs and coupled spikes suggests a direct synaptic interaction. We tested whether we could recapitulate the coupling probability of spontaneously active neurons by direct stimulation of the propagation signal neurons. In this example, the coupled eAPs at electrode A7 (Fig. 3a) consistently and closely followed the propagation signal D3/E5, with a probability of 0.65 (n = 377 coupled spikes). If eAPs from the propagation signal D3/E5 result in spikes at A7, stimulation of electrode D3 should result in eAPs that occur with a similar probability and amplitude distribution at A7. As shown, stimulation resulted in eAPs in A7 with a probability of 0.79 (n = 393 coupled spikes; Fig. 3a). The spike amplitudes at A7 following spontaneous propagation signals (44.14 ± 9.15 µV) or stimulation (44.75 ± 14.17 µV) did not differ (n = 200 randomly chosen spikes from each group; *P* = 0.2; two-sample KS test, two-sided; Fig. 3b). The latency of the coupled spikes was 4.29 ± 0.69 ms in the spontaneous conditions and 5.93 ± 0.63 ms in the stimulated condition. For stimulation, the latency was the interval between the beginning of the blanking period and the negative peak of the coupled spike. The longer latency following stimulation likely reflects the difference in how latency was measured in these cases. This suggests that spontaneous eAPs at A7 following the propagation signal or the stimulation of the propagation signal are from the same source neuron.

Across multiple experiments, the coupling probability between spontaneous and stimulated cases were comparable, as shown by plotting coupling probability for spontaneous events as a function of coupling probability for evoked events (Fig. 3c). The slope of the linear fit to this data was 0.84 (n = 20). A paired t-test (two-sided) showed no significant difference when comparing coupling probability for spontaneous and evoked cases (*P* = 0.45). Similarly, a plot of the mean amplitude of spontaneous eAPs as a function of the amplitude of evoked eAPs was well fitted to a line with a slope of 0.93; the amplitudes of coupled spikes following stimulation were not statistically different from those of spontaneous spikes (Fig. 3d; n = 20; *P* = 0.18; paired t-test, two-sided). Our experiments directly demonstrate that the spike clusters seen following many propagation signal spikes result at least in part from activation of synapses originating from the single neuron identified by its propagation signal. These data indicate that stimulation at the electrode where a propagation signal was detected recapitulates the probability and amplitude characteristics of spontaneous events that resemble coupled spikes.

### Decreasing active synapse density lowers coupling probability

**Figure 4:**
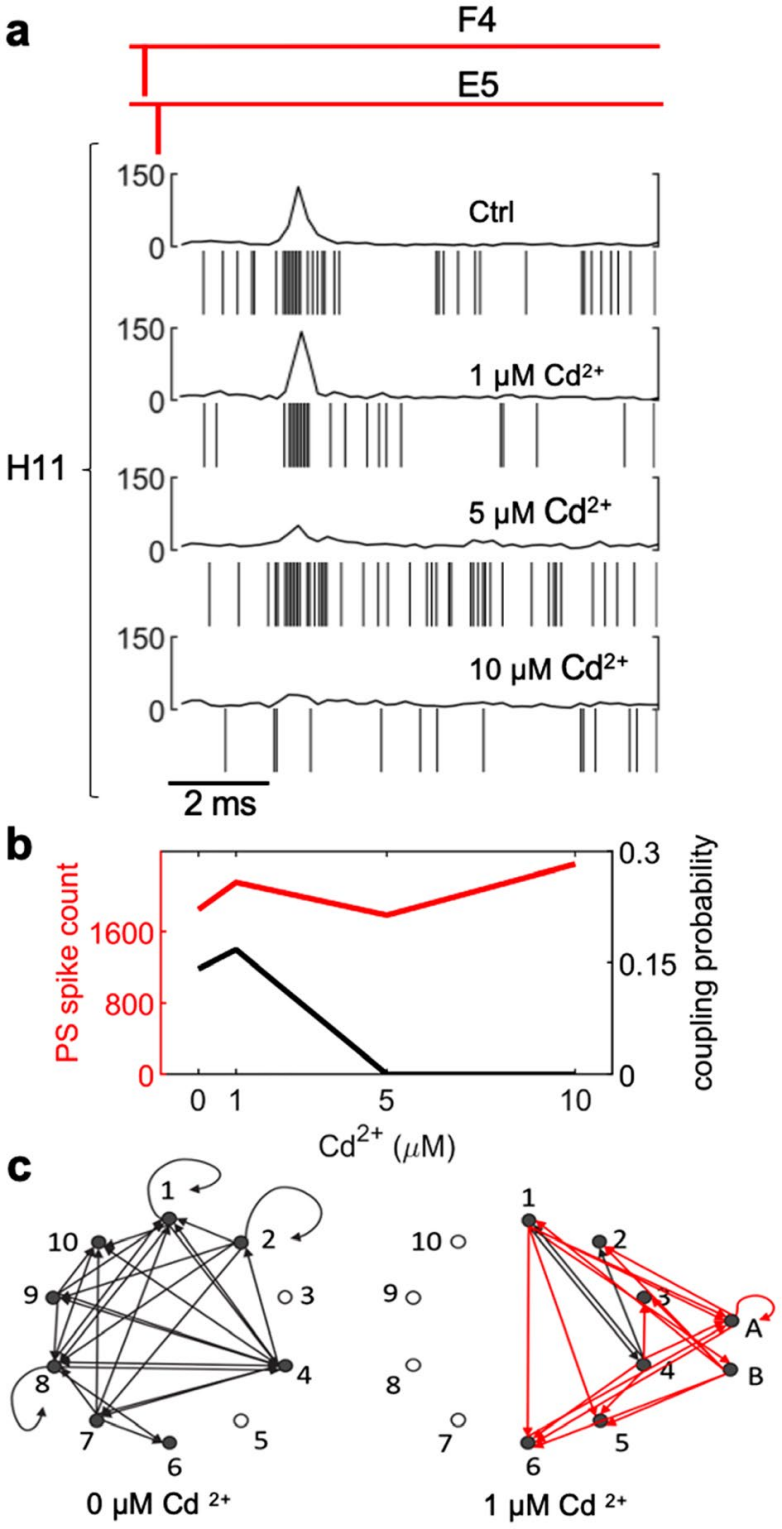
Cadmium alters coupling probabilities. (**a**) Propagation signal eAP’s detected at F4 and E5 with coupled spikes in H11 in the absence of cadmium and after addition of 1 µM, 5 µM, and 10 µM cadmium. Coordinated spiking activity in H11 after propagation signal spikes in F4 and E5 decreased dramatically and resulted in the absence of coordinated activity at 10 µM. (**b**) In this example, the total number of spikes from the propagating neuron detected in electrode F4 to E5 under control conditions was 1848 spikes and the coupling probability of the postsynaptic unit at H11 was 0.14. Addition of 1 µM Cd^2+^ resulted in 2154 spikes in the propagating neuron and a coupling probability of 0.16 with H11. At 5 µM and 10 µM Cd^2+^, the spikes CCG at electrode H11 did not meet our criteria for coupling and were considered fully de-coupled; with 1784 and 2357 spikes in the propagating neuron, respectively. (**c**) Network graphs were constructed to visualize the couplings between only propagation signal spiking activity. 8 of 10 propagation signals (nodes) formed a total of 29 couplings (black edges) in control conditions. Addition of 1 µM Cd^2+^ resulted in loss of 26 edges present in control and the appearance of 17 couplings (red edges). No couplings were detected at 5 µM or 10 µM Cd^2+^ (1 MEA was used for 4 recording sessions for **a**–**c**).

Presynaptic calcium influx initiates the release of neurotransmitters^8^. Cadmium (Cd^2+^) reduces calcium conductance through calcium channels^9^ and decreases the neurotransmitter release probability, and thus the number of active synapses^10^. However, the number of synapses between individual cultured hippocampal neurons can be sufficiently high such that single axons can make multiple synapses on the dendrites of single postsynaptic neurons^11,12^. If coupled spikes result from integration of multiple synaptic inputs from single axons, then decreasing the number of active synapses with Cd^2+^ should decrease the coupling probability. We compared the coupling probabilities in control conditions and following the addition of Cd^2+^ (1 µM, 5 µM and 10 µM) in the same cultures. The majority of cases resembled the coupling between propagation signal F4/E5 and postsynaptic electrode H11, in which coupled spikes in control conditions were no longer present at 10 µM Cd^2+^ (Fig. 4a). We identified 83 functional couplings in control which decreased to 67 at 1 µM Cd^2+^. Higher Cd^2+^ concentrations decreased the detection of coupling to 18 (5 µM Cd^2+^) and 10 (10 µM Cd^2+^). Interestingly, we saw no change in latency between control (2.9 ± 1.22, n = 83) and 5 µM Cd^2+^ (2.8 ± 0.77, n = 18). The total number of propagation signal spikes were comparable in all conditions (Fig. 4b), indicating that the cadmium-related decrease in coupling probability was unrelated to changes in number of propagation signal spikes, and thus neuronal activity. These results demonstrate that the coupling probability is sensitive to changes in the number of active neurotransmitter release sites.

To examine how decreasing the number of active synapses alters the connectivity of small neural networks, we used a recording that demonstrated a high level of coupling among propagation signals and asked how reduction of active synapses is affected with 1 µM Cd^2+^. Propagation signal neurons are represented by circles (nodes) in the network graph (Fig. 4c), and lines (edges) represent couplings between propagation signals. We identified 29 couplings (black edges) among 8 of 10 of the propagation signals in control conditions, including 3 recurrent connections. With the addition of Cd^2+^ only 3 of the 29 couplings present in control were retained (black lines, Fig. 4d, right). Interestingly, addition of Cd^2+^ revealed 17 couplings not seen in control conditions, including couplings among propagation signals that were only detected in Cd^2+^. In this experiment, no couplings were detected in 5uM Cd^2+^. The decrease in network connectivity in Cd^2+^ is consistent with reduction in the number of active synapses. Cd^2+^ also affects inhibitory neurons, and the consequent reduction in inhibitory tone may explain the redistribution of connections among existing and new propagation signals.

### Temperature sensitivity of coupled eAP latency

**Figure 5:**
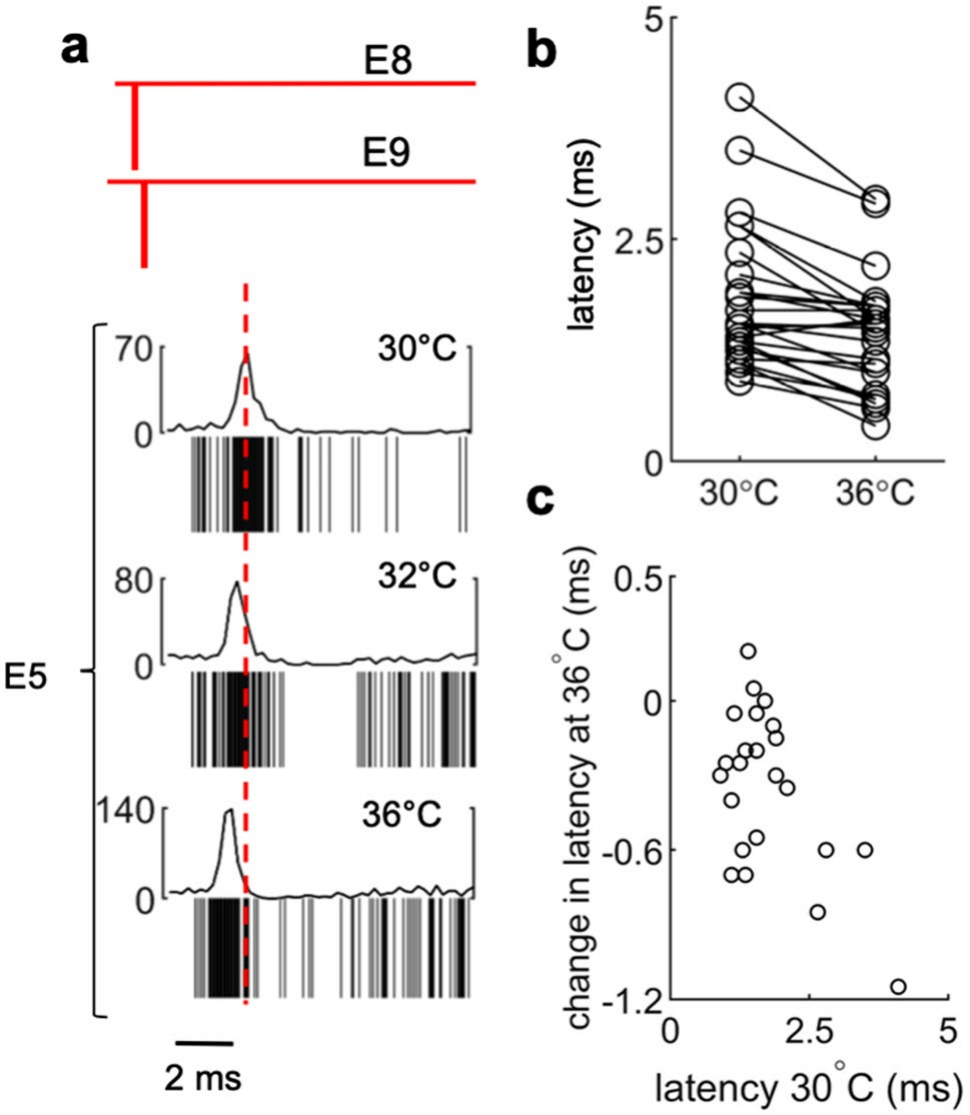
Temperature increase reduces coupling latency. (**a**) Spikes from a propagating neuron detected at E8 and E9 coupled to a postsynaptic unit in E5. At 30 °C the average latency between propagation signal spikes and the CCG peak in E5 was 2.75 ± 0.47 ms. Temperature in the same culture was increased to 32 °C and 36 °C sequentially, the average latency of the CCG peaks were 2.15 ± 1.09 ms and 1.80 ± 0.91 ms, respectively (1 MEA was used over 3 recording sessions for (**a**). (**b**) The distribution of average latency of presynaptic propagation signals coupled to postsynaptic units at 30 °C is significantly different than the average latency of the same couplings at 36 °C (1.76 ± 0.8 ms at 30 °C, 1.41 ± 0.7 ms at 36 °C; n = 23; *P* < 0.0001, paired t-test, two-sided). (**c**) Average latency of couplings at 30 °C versus the change in average latency of the same couplings at 36 °C. (7 MEAs were used with 2 recording sessions per MEA, 14 total sessions, for (**b**) and (**c**)).

Synaptic transmission is highly sensitive to temperature^13–15^. If postsynaptic activation and integration of multiple synapses from single axons is the basis for the eAP coupling latency, then changes in the coupling latency in response to temperature should be within range of previous measurements of synaptic delay^16–18^. Thus we measured the latency between propagation signal spikes and coupled spikes at three temperatures. As shown (Fig. 5a), increasing the temperature shifted the distribution of the coupled eAP latency to shorter values in this example (2.75 ± 0.47 ms at 30 °C; 2.15 ± 0.1.09 ms at 32 °C; 1.80 ± 0.91 ms at 36 °C). The mean latency at 30 °C (1.76 ± 0.8) was longer than at 36 °C (1.41 ± 0.7; n = 23; *P* < 0.0001 paired t-test; Fig. 5b). The coupling latency captures the sum of events that occur between spiking in one neuron to spiking in another, including action potential propagation, the synaptic delay and synaptic integration. At physiological temperatures, the synaptic delay at mammalian synapses can range from 150 to 500 microseconds and can decrease by 50% within the temperature range of our experiments^17,18^. Action potential propagation velocity increases by 30% between 30 and 36 °C ^4^. Plotting the change in coupling latency at 36 °C as a function of the latency at 30 °C (Fig. 5c) highlights the variability of the latency change in our data set. However, the mean coupling latency change between 30° to 36 °C (0.35 ± 0.32 ms, n = 23) is consistent with expected differences in temperature dependence of synaptic delay and action potential propagation. Interestingly, shorter coupling latencies at 30 °C tended to have smaller temperature dependent changes, suggesting that these examples are close to the lower limit of our temporal resolution.

### Contributions of multiple presynaptic neurons to spiking in a single postsynaptic neuron

**Figure 6:**
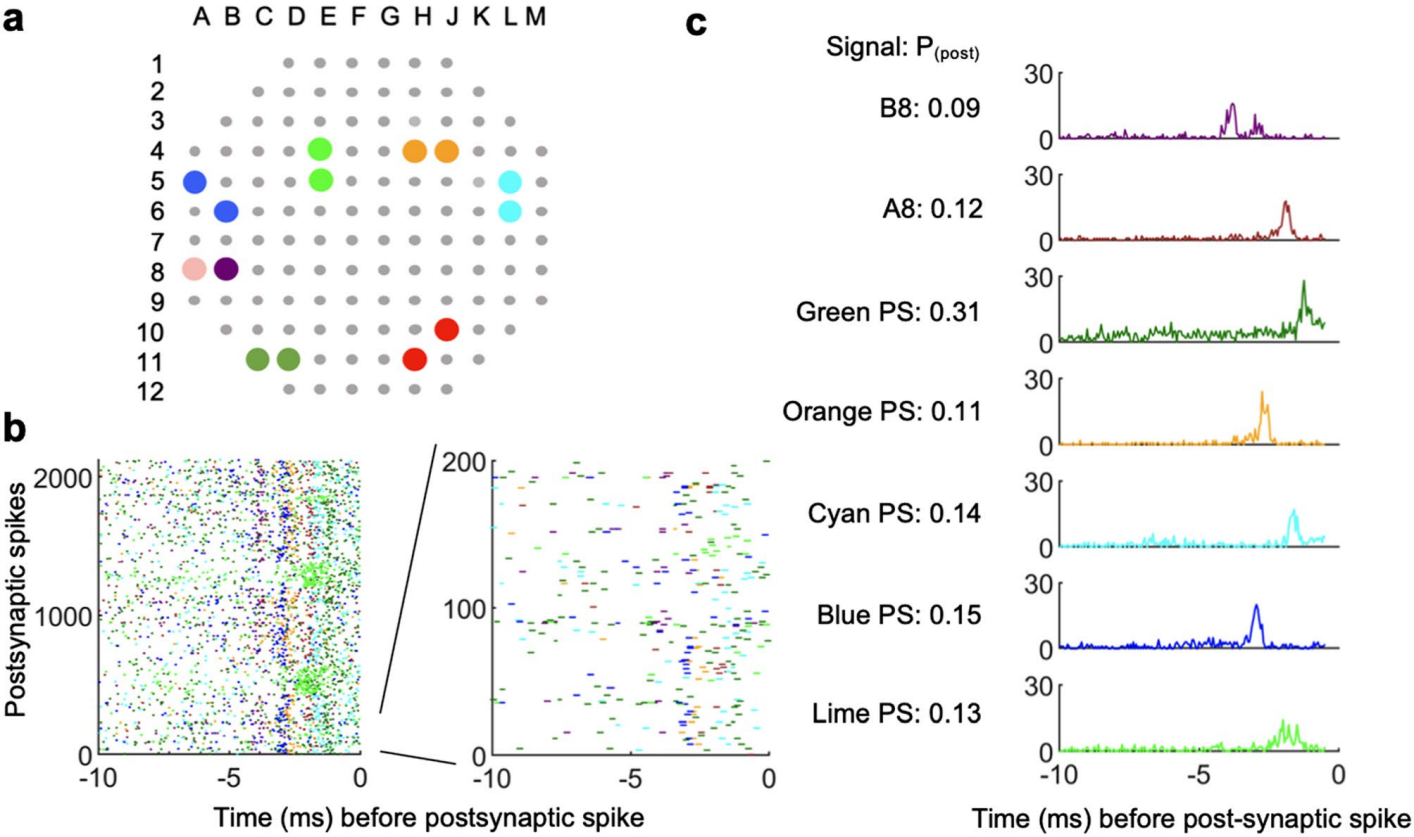
The influence of presynaptic inputs on a single postsynaptic propagation signal. (**a**) Location of the post-synaptic neuron in red and upstream signals in other colors. Dots in different colors represent different upstream signals. Each two dots in the same color represent two anchor points for one propagation signal. Pink and purple represent two single electrode units. (**b**) The patterns of pre-synaptic spikes. Each row shows the pre-synaptic firing pattern within 10 ms prior to corresponding spike on the red neuron. The first 200 instances are zoomed in on the right. The colors are consistent with (**a**). (**c**) CCGs for all upstream signals. Postsynaptic spikes (red propagation signal, n = 2124 spikes) were used as reference time points to perform CCG on other propagation signals and single electrodes A8 and B8. Because the postsynaptic spikes were used as the reference time points, the coupling probability was calculated by taking the ratio of presynaptic spikes in the CCG peak over the total number of postsynaptic spikes (1 MEA and a single recording session was used for (**a**–**c**)).

The coupled eAPs we identified likely represent synaptic couplings. However, it is unlikely that activation of synapses from the majority of single axons reflects the most common source of action potentials in postsynaptic cells, given that most of our measurements of coupling probability were well below one. To address how multiple inputs influence a postsynaptic neuron, we used spike times from a single propagation signal to identify presynaptic couplings. In one example, we identified 7 inputs that were statistically coupled to the propagation signal eAPs. Of these, 5 inputs were propagation signals, and 2 were isolated as spikes from single electrodes (Fig. 6a). A raster display of eAPs from these 7 inputs highlights that the majority of presynaptic spikes occur 1–5 ms before the postsynaptic propagation signal eAPs (Fig. 6b). For each presynaptic input, the probability of a spike occurring 0.5–10 ms before the postsynaptic propagation signal eAP was computed as the ratio of the amount of post-synaptic spikes that has at least one presynaptic spike in the 9.5 ms window over the total number of post-synaptic spikes. The coupling probabilities of the 7 inputs ranged from 0.09 to 0.31 (Fig. 6c). The low electrode density likely means that we do not capture all neurons presynaptic to the propagation signal neuron. Additionally, the relatively low coupling probabilities likely reflect the necessity for multiple well-timed co-active presynaptic inputs to bring a postsynaptic neuron to AP threshold.

## Discussion

In this work we present analytical approaches for detecting when synapses from single axons influence the ability of postsynaptic neurons to fire action potentials. Detection of action potential propagation validates that the preceding, presumably presynaptic, eAPs arise from single identifiable neurons. The timing of these eAPs is the clock we use to detect short latency spike clusters that follow, or even precede, propagating spikes. Direct stimulation of presynaptic neurons validated our assertions. As expected, decreasing the number of active synapses with cadmium decreased the coupling probability. Given the timing and latency characteristics of what we define here as functional coupling, our data is most consistent with functional coupling representing the action of multiple synapses from a single axon acting on a single postsynaptic neuron in many cases. We cannot rule out that some of what we are detecting represents indirect synaptic coupling but for that to explain our data, the latencies and fidelity would have to be higher than expected from the literature. Our in vitro results are consistent with work in well characterized in vivo circuits^19,20^. With this in vitro system we can screen the effects of pharmacological manipulations as well as the synaptic connectivity phenotypes from targeted mutant or iPS-derived neurons.

Techniques such as simultaneous recordings with multiple patch clamp electrodes^21,22^, a combination of patch and extracellular recording^23,24^ and automated multi-patch recording^25^ leave no doubt about the neuronal source of activity. However, the technically challenging nature of these methods makes their routine use difficult. The invasive nature of patch recording also precludes long-term monitoring of neurons. The results from our experiments would be difficult to obtain with other methodologies such as optical imaging methods that require fluorescence indicators resulting in phototoxicity and altered cell physiology^26^. By unambiguously isolating eAPs from single identifiable neurons, our methods demonstrate how synaptic coupling from multiple identified neurons can be simultaneously assessed, under a variety of experimental manipulations and/or across multiple days. High signal fidelity and temporal resolution with the MEA, capable of capturing spike amplitudes while recording from multiple sites, allowed us to infer a partial connectivity map for the culture (Fig. 4d). This “functional connectome” is clearly missing connectivity edges that are invisible due to the low electrode density relative to the density of the neurons and the presence of neurons outside the boundary of the array. However, another source of missing connectivity can be revealed when synaptic weights are changed as implemented by reducing the release probability (Fig. 4d). Reducing the number of active synapses with cadmium resulted in the expected loss of connections, but also the emergence of new connections, a latent connectivity network, probably due to suppression of inhibitory transmission. Likewise, detection of multiple inputs to a single neuron and determining each of their latencies and coupling probabilities (Fig. 6) presents novel approaches to dendritic signal summation^27,28^. Our data revealed a multiplicity of input firing events within a 5 ms window that contribute, with different probabilities, toward eliciting a post synaptic action potential.

## Methods

### Neuronal cell culture

The animal protocols and procedures in this study were approved by the Institutional Animal Care and Use Committee (IACUC) of University of California, Santa Barbara and were performed in accordance with the National Institutes of Health Guide for the Care and the Use of Laboratory Animals. All animal experiments were performed in accordance with ARRIVE guidelines.

We prepared hippocampal neurons from postnatal day 0 (P0) C57BL/6 male mice using a previously described protocol^29^. Up to 3 animals were used per experiment and the pooled hippocampal neurons were plated on multiple MEAs. Cleaned and sterilized multi-electrode arrays (120MEA100/30iR-ITO arrays; Multi Channel Systems) were coated with 0.1 mg/ml poly-L-lysine (Sigma-Aldrich) for 1 h at 37 °C, rinsed 3 times with sterile water and air dried before plating. Plating was done in two steps. In the first step, cultured glial cells maintained in separate T-75 flasks were dissociated and plated (at 150,000 cells per well) on MEAs and allowed to proliferate. Once glia were confluent over the electrode area, freshly dissociated hippocampal cells were plated at 250,000 cells per dish (550 cells/mm^2^) on the confluent glial cultures. Cultures were grown in minimum essential medium with Earle’s salts (Thermo Scientific, catalog # 11090081) with 2 mM Glutamax (Thermo Scientific), 5% heat-inactivated fetal bovine serum (Thermo Scientific), and 1 ml/l Mito + serum extender (Corning) and supplemented with glucose to an added concentration of 21 mM. For the purpose of long-term culturing and maintaining MEA sterility during recordings, the MEA chamber was covered with a membrane that permits CO_2_ exchange when the plate is in the CO_2_ incubator and during recordings.

### MEA recordings

Extracellular voltage recordings of neuronal cultures were performed using an MEA 2100-System (Multichannel Systems, Reutlingen, Germany). Arrays contained 120 electrodes with a 100 µm inter-electrode distance. Voltage records were acquired at 20 kHz. All recordings were performed in culture media. The head stage temperature was set to 30 °C with an external temperature controller and MEAs were equilibrated for 5 min on the head stage before data acquisition or after any pharmacological or temperature manipulation. Recording duration was typically 3 to 5 min. Only cultures at 14 days in vitro (DIV) or older were used for all pharmacological, stimulation and temperature experiments. These experiments used pooled neurons from up to 3 animals.

### Data processing

Raw extracellular voltage data from Multichannel Systems acquisition software was converted to HDF5 file format using Multichannel Data Manager software and processed offline. Using MEA tools^30^, extracellular voltage records were bandpass filtered using a second order Butterworth filter with cutoff frequencies of 200 Hz to 4000 Hz followed by spike detection. Negative deflections in the voltage records were labelled as spikes when the amplitude exceeded 6 times the standard deviation of the median noise level. None of the data in this work was spike sorted. Spike times and amplitudes output from MEA Tools were used for subsequent analysis and development of automated algorithms in Matlab. Post-hoc analysis was performed in Matlab by extracting the raw voltages for electrodes of interest and inspecting the timing and amplitude of spikes within time windows of interest. In certain cases, to validate our results we shuffled spike times from electrodes of interest; spike time shuffling was done in a way that retained the inter-spike interval distribution from the spike train of interest to retain the overall spiking pattern. Propagation signals were differentiated from coupled signals based on the difference in inter-electrode latency. The inter-electrode latency of the propagation signals we detected had a mean of 0.46 ± 0.36 ms (n = 1014 electrode pairs). In contrast, the latencies of what we detected as coupled neurons had a mean of 2.79 ± 2.06 ms, (n = 746) in wild type neurons.

### An algorithm for automated detection of neuronal coupling

To facilitate identification of coupled spiking between two neurons we developed a set of algorithms for the automated detection of (1) propagation signals and (2) functionally coupled units (available at https://github.com/ZhuoweiCheng/Propagation-Signal-and-Synaptic-Coupling-Algorithm). For propagation signal detection, spike times were stored for all 120 electrodes in a cell array. The set of electrodes was denoted as *E*. To identify all propagation signals in an array, each electrode *e*_*i*_ $\in$ *E* was used as a reference electrode to compare with all electrodes *e*_*j*_ (*j* = 1, 2, …, n). Cross-correlograms (CCGs) were constructed using a 2 ms window before and after reference time-points for each (*e*_*i*_, *e*_*j*_) pair. Let n_all_ denote the number of spikes on the reference electrode and n_win_ denote the largest sum of counts in any 0.5 ms moving window in the cross-correlogram (CCG). A greater than 0.3 ratio indicates a consistent spike time delay of the target electrode with respect to the reference electrode. The ratio 0.3 was determined empirically with results from different thresholds compared with manually detected propagation signals. If a high ratio was detected in the CCG for (*e*_*i*_, *e*_*j*_), the delay time of *e*_*j*_ corresponding to *e*_*i*_ is recorded. All electrodes with high ratios were sorted based on their delay time. If all *e*_*j*_ have a non-negative delay time, then we can conclude that a propagation signal originating from *e*_*i*_ was detected (Fig. 2a). The process was repeated for all electrodes in the array generating a collection of propagation signals.

We then used the set of the identified propagation signals, denoted as *S*, to find instances of putative intercellular coupling. The putative functionally coupled relationships between all *s*_*i*_ $\in$ *S* or between *s*_*i*_ $\in$ *S* and *e*_*j*_ $\in$ *E* can be identified. To identify connections, we began with spike times from individual propagation signals. Spike times *T*_*i*_ for a propagation signal were computed using the spike times of the group of electrodes. Let *e*_*1*_ denote the earliest electrode on a signal and *e*_*x*_ denote the electrode with the most spikes other than *e*_*1*_. The spike times, *T*_*i*_, are then computed with the average times of the co-occurrences in these two electrodes. Co-occurrences are defined as two spikes occurring in quick succession in separate electrodes.

We filtered our results as follows: (1) to minimize false-positive couplings, the lower limit of coupling probability was set to 0.1, (2) couplings with an average latency outside our window of interest (1–5 ms after the propagation signal eAP) were discarded as most central nervous system excitatory neurons couple to neighboring neurons with a latency within this range^31,32^, and (3) the area under the CCG peak must be 60% of the total area to avoid chance associations between pre- and postsynaptic spikes. To accomplish this, each signal *s*_*i*_ was used as a reference signal to compare with all other signals *s*_*k*_ (k ≠ i) or all electrodes *e*_*j*_. A CCG, using a window between 0.5 and 10 ms after reference time-points were performed for each (*s*_*i*_, *s*_*j*_) or (*s*_*i*_, *e*_*j*_) pair. The criteria for identifying connections are:

1. If *n*_*1*_ denotes the sum of counts of the CCG and *n* denotes the number of spikes in the reference signal, the ratio *n*_*1*_/*n* must be larger than *v*_*1*_.
2. If *n*_*2*_ denotes the largest sum of counts in any 3 ms moving window in the CCG, then the ratio *n*_*2*_/*n*_*1*_ must be larger than *v*_*2*_.
3. The delay time must be between *v*_*3*_ and *v*_*4*_ ms.
4. The standard deviation for all the Δt in CCG is less than *v*_*5*_.

The values used in this paper are *v*_*1*_ = 0.1; *v*_*2*_ = 0.57; *v*_*3*_ = 1; *v*_*4*_ = 5; *v*_*5*_ = 2.7. Specifically, v_2_ and v_5_ were determined empirically. The value v_2_ is a cutoff for the ratio of the number of spikes in the 3 ms moving window with the most spikes to the total number of spikes in the CCG. The larger the value of v_2_, the stricter the criteria and the higher the certainty that the coupling results identified by the algorithm are real and non-random. However, the higher the value is for v_2_, the more likely we are to exclude real couplings. The value v_5_ is a threshold of the standard deviation of the latency. We used v_5_ to filter out cases where one electrode detects eAPs from multiple nearby neurons, as can often happen in our recordings (data not shown). Between two electrodes, the coefficient of variation of eAP propagation latency should be small. It was often the case that single electrodes detected spikes from multiple neurons (for example Figs. 1f and 2d) at the neuron densities we used. When spikes from multiple independent neurons are detected at any single electrode, the latency distribution of spikes between the target electrode and the reference electrode is expected to have large variability. All *v*_*i*_ can instead be user defined. If the relationship is between propagation signals and spikes at individual electrodes, the output of the algorithm also includes a verification flag. A flag value of one is a suggestion to manually verify the connection. Flag values are determined by comparing the normalized standard deviation of the voltage amplitude of all spikes on the target electrode to v_6_. The value used in this paper for v_6_ is 0.25.

### Statistical analysis

Statistical analysis was performed with Prism 8 or Matlab. Distributions were tested for normality using a one-sample Kolmogorov–Smirnov test. Non-uniformly distributed data were tested using non-parametric tests (two-sample Kolmogorov–Smirnov test or Mann–Whitney U-test) at a 5% significance level.

## Data Availability

All data are available from the corresponding author upon reasonable request.

## Code availability

Custom Matlab code is available at https://github.com/ZhuoweiCheng/Propagation-Signal-and-Synaptic-Coupling-Algorithm.
